# Biometal Dyshomeostasis and Toxic Metal Accumulations in the Development of Alzheimer’s Disease

**DOI:** 10.3389/fnmol.2017.00339

**Published:** 2017-10-24

**Authors:** Yong Li, Qian Jiao, Huamin Xu, Xixun Du, Limin Shi, Fengju Jia, Hong Jiang

**Affiliations:** ^1^Shandong Provincial Key Laboratory of Pathogenesis and Prevention of Neurological Disorders and State Key Disciplines: Physiology, Department of Physiology, Medical College of Qingdao University, Qingdao, China; ^2^Shandong Provincial Collaborative Innovation Center for Neurodegenerative Disorders, Qingdao University, Qingdao, China

**Keywords:** Alzheimer’s disease, biometals, metal transporters, neurotoxicity, protein aggregation, dementia

## Abstract

Biometal dyshomeostasis and toxic metal accumulation are common features in many neurodegenerative disorders, including Alzheimer’s disease (AD), Parkinson’s disease, and Huntington’s disease. The neurotoxic effects of metal imbalance are generally associated with reduced enzymatic activities, elevated protein aggregation and oxidative stress in the central nervous system, in which a cascade of events lead to cell death and neurodegeneration. Although the links between biometal imbalance and neurodegenerative disorders remain elusive, a major class of endogenous proteins involved in metal transport has been receiving increasing attention over recent decades. The abnormal expression of these proteins has been linked to biometal imbalance and to the pathogenesis of AD. Here, we present a brief overview of the physiological roles of biometals including iron, zinc, copper, manganese, magnesium and calcium, and provide a detailed description of their transporters and their synergistic involvement in the development of AD. In addition, we also review the published data relating to neurotoxic metals in AD, including aluminum, lead, cadmium, and mercury.

## Introduction

Alzheimer’s disease (AD) is the most common neurodegeneration disorder linked with dementia in the elderly (Todd et al., 2013). Neuropathological changes in the AD brain are related to the aggregation of amyloid-beta (Aβ) peptide which forms senile plaques and initially leads to a series of consequences including hyperphosphorylated aggregates of the microtubule-associated tau protein in neurofibrillary tangles (NFTs), altered neuronal connectivity and neuronal loss (Tanzi and Bertram, 2005). Over the past two decades, the structure of Aβ and its toxic roles on the induction of oxidative stress, neuroinflammation and autophagy, have been extensively studied (Jomova et al., 2010). Furthermore, several drugs have been established for the treatment of AD, which involve the obliteration or reduction of Aβ production, however, most of these treatments failed during their respective clinical trial phases (Ayton et al., 2015). More recently, new challenges have emerged in that the aggregation of Aβ in the pathogenesis of AD is now thought not to represent an initial event, but rather a subsequent event of the disease (Kepp, 2017). Therefore, the exploration of new research directions for the treatment of AD has become very important. There is considerable evidence to suggest that the homeostasis of essential biometals (e.g., iron, zinc, copper, manganese, magnesium, and calcium) is disrupted in AD, and that these metals play an important role in the aggregation and metabolism of Aβ and tau protein. Based on this, researchers have proposed a metal hypothesis for AD (Bush and Tanzi, 2008; Ayton et al., 2015), which gives rise to the notion that targeting metal interactions with Aβ might be more effective in preventing the disease.

The pathophysiological roles of metal imbalance in the brain have been recently described in several outstanding reviews (Chin-Chan et al., 2015a; Zhang et al., 2016). However, the dysregulation of biometal function as a cause of AD is still a matter of debate. Since biometals cannot passively pass through the blood–brain barrier (BBB), the described metal imbalance in the AD brain cannot merely be related to the increased or decreased exposure to metals, but rather to a more primary distribution of intracellular ions in a confusing way. Thus, the homeostasis of brain metals controlled by various metal importers, exporters, and metal sequestering proteins in a specific disease is of particular interest. Recently, the dysregulation of these metal transport-related proteins in the pathogenesis of AD has been extensively studied. Therefore, in this review, we describe the recent advances in our understanding of the role of biometals in the molecular mechanisms underlying AD. Furthermore, we provide a detailed description of the distinct abnormal regulation of biometals in AD, focusing particularly on the role of their correlative transporters in these processes. Finally, we present evidence of molecular links between toxic metals and AD.

## Biometals and their Transporters

### Iron

Iron is an essential transition metal for many fundamental neuronal functions in the brain, such as oxygen transport, mitochondrial respiration and myelin synthesis, as well as acting as a cofactor for a large number of metalloproteins involved in metabolism and signal transduction (Castellani et al., 2012; Biasiotto et al., 2016). Iron deficiency affects numerous neurological peculiarities, especially during babyhood, causing brain development stagnation. Iron levels in the brain increase with aging; however, excessive levels of iron lead to an over-abundant production of reactive oxygen species through Fenton chemistry, which eventually causes cell damage (Droge and Schipper, 2007; Muhoberac and Vidal, 2013). Moreover, a growing number of studies have reported that disruption of iron homeostasis may be a hallmark of many neurodegenerative disorders (Ward et al., 2014; Jiang et al., 2017).

Many studies, using a variety of different techniques, have identified that iron levels are increased in AD brains (Bartzokis et al., 1994; LeVine, 1997), specifically in the globus pallidus and putamen (Wang et al., 2014; Moon et al., 2016). However, meta-analyses have revealed unchanged or reduced serum iron levels in AD patients with respect to healthy subjects (Tao et al., 2014; Wang et al., 2015). Although the explanation for this imbalance is still unknown, studies on the role of iron in AD have shown that excessive iron stimulates hydroxyl radical formation via the Fenton reaction, which may contribute to increased oxidative stress levels in AD. Oxidative damage to AD-linked protein aggregations by excessive levels of iron can cause synaptic dysfunction and neuronal cell death, a detrimental process which can be alleviated by the administration of iron chelators (Salkovic-Petrisic et al., 2015). High iron content is loaded around senile plaques and elevates the production of Aβ by increasing the expression of amyloid-β precursor protein (APP). Iron modulates APP transcription through iron-responsive elements (IREs) present in the 5′-untranslated region of APP mRNA (Rogers et al., 2002). Excessive iron levels result in the dissociation of iron regulatory proteins (IRPs) from their IRE binding sites and abolish their repression of APP mRNA, leading to increased APP translation, whereas lead (Pb) represses the IRE-mediated enhancement of APP translation (Rogers et al., 2016). Furthermore, iron has a high affinity for binding with Aβ *in vitro*, which could promote its aggregation and accelerate the formation of oligomers (Honda et al., 2004; Liu et al., 2011). Previous studies have reported the presence of Aβ reduced reactive oxygen species (ROS) at the beginning of neurodegeneration, which probably benefit from the alleviation of oxidative stress by binding iron (Nunomura et al., 2001; Jolivet-Gougeon and Mallet, 2016). However, a high iron concentration gives rise to the antioxidant system becoming overburdened and thus results in cellular damage over time, leading to a vicious cycle involving a higher production of Aβ as a result of enhanced oxidative stress (Tamagno et al., 2008). In addition to Aβ, iron also binds to tau, which promotes its aggregation in iron-enriched regions (Sayre et al., 2000). Similarly, iron has also been shown to induce tau phosphorylation, which might be caused by the activation of cyclin-dependent kinase 5 (CDK5) and glycogen synthase kinase 3β (GSK3β) pathways (Lovell et al., 2004). The administration of iron chelators, such as desferrioxamine, could reduce iron-induced tau phosphorylation in AD transgenic mice (Guo et al., 2013).

**FIGURE 1:**
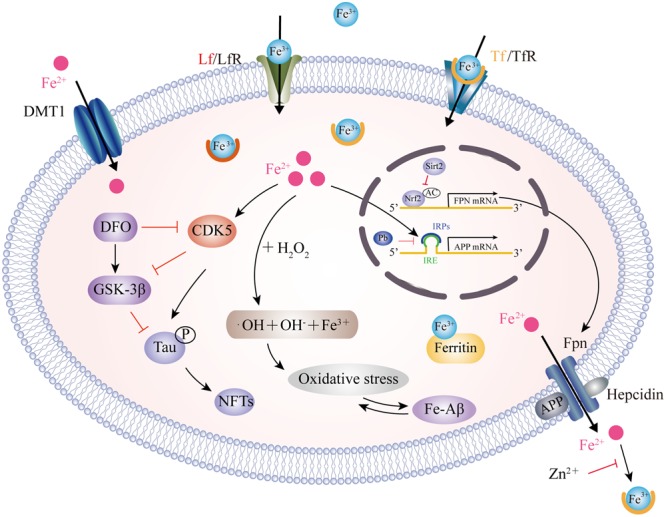
Model for iron transport in neurons and iron imbalance associated with AD. Two pathways have been well-documented for cellular iron uptake: TfR endocytosis-mediated Tf-Fe^3+^ entry and the direct import of Fe^2+^ by DMT1. The only way for iron to be transported out of the cell is by Fpn with the ferroxidase activity of CP or HP. However, hepcidin binding to Fpn causes its internalization to prevent Fe^2+^ export. Intracellular Fe^2+^ binds to the IRE in the 5′ UTR of APP mRNA, which reverses the repression of IRP1 and promotes APP translation. Pb enhances the IRP1/IRE-mediated repression of APP. APP also has ferroxidase activity and interacts with Fpn to oxidize Fe^2+^ into Fe^3+^ for Tf binding. APP ferroxidase is inhibited by extracellular Zn^2+^. In addition, sirt2 regulates Fpn expression via the deacetylation of Nrf2, which in turn controls cellular iron homeostasis. In the AD brain, increased levels of Fe^2+^ induces the Fenton reaction to produce ^•^OH, resulting in oxidative damage and Aβ production. In addition, Fe^2+^ can bind to Aβ and enhance its aggregation, while Fe^2+^ increases tau phosphorylation via activation of CDK5 and GSK3β. This process can be inhibited by DFO administration. CP, ceruloplasmin; HP, hephaestin; IRE, iron responsive element; IRP1, iron regulatory protein; Pb, lead; Sirt2, sirtuin 2; Nrf2, nuclear factor erythroid-derived 2-related factor 2; CDK5, cyclin-dependent kinase 5; GSK3β, glycogen synthase kinase 3β; DFO, desferrioxamine.

A dynamic balance between iron influx and efflux is critical for intracellular iron homeostasis, in which multiple transporter proteins play key roles. The dysregulation of iron importers, including transferrin (Tf), divalent metal transporter 1 (DMT1), lactoferrin (Lf) and melanotransferrin (MTf), as well as iron exporter ferroportin (Fpn), may account for iron accumulation in the affected brain regions of AD patients (**Figure 1**). DMT1, also known as divalent cation transporter 1, is expressed on neurons, astrocytes and microglia but not oligodendrocytes, and is relevant to the pathway involved in the influx of Fe^2+^ (Song et al., 2007). Two isoforms of DMT1, DMT1-IRE and DMT1+IRE, have been shown to colocalize with Aβ in the plaques of the AD brain and the levels of both DMT1 isoforms are significantly increased in the frontal cortex and hippocampus in a APP/PS1 transgenic mouse model (Zheng et al., 2009), which was accompanied by a reduction in Fpn expression (Xian-hui et al., 2015), suggesting that the deregulation of iron metabolism-related protein DMT1 and Fpn plays a critical role in the iron-mediated neuropathogenesis of AD. Hepcidin, an iron-homeostatic peptide, is colocalized with Fpn in neurons and astrocytes showed a reduced expression of Fpn in AD brains. Downregulation of hepcidin exhibits impairment in the iron export pathway which results in cellular iron retention (Raha et al., 2013). APP is a transmembrane protein which was found to catalytically oxidize Fe^2+^ to Fe^3+^ by ferroxidase activity and then interact with Fpn to promote iron export (McCarthy et al., 2014); however, this process is inhibited by extracellular zinc, which originates from zinc-Aβ complexes (Duce et al., 2010). From the same laboratory, these authors also showed that loss of soluble tau could cause iron retention by impairing APP-mediated iron export (Lei et al., 2012), and that such suppression can be caused by an iron chelator (Lei et al., 2015) or by lithium treatment (Lei et al., 2017). Furthermore, sirtuin 2 was reported to regulate cellular iron homeostasis via the deacetylation of nuclear factor erythroid-derived 2-related factor 2, which acts as a transcription factor to control Fpn expression (Yang et al., 2017). Interestingly, a recent report showed that compounds extracted from Chinese herbs could down-regulate DMT1 expression and up-regulate Fpn expression, thus providing a new strategy for reducing iron overload-induced impairment in AD (Dong et al., 2015). The Tf-transferrin receptor (TfR) complex is responsible for iron uptake in endothelial cells of the BBB. The transport of iron across this barrier is most likely the result of the receptor-mediated endocytosis of Tf-bound iron (Moos and Morgan, 2000). A previous proteomics study found that Tf levels were significantly different in the cerebrospinal fluid (CSF) of familial AD when compared between those who carried mutations and related non-carriers (Ringman et al., 2012). The structure of Lf is similar to Tf, in that they are both composed of two lobes, each having one binding site for Fe^3+^ (Baker et al., 1994). Lf is highly expressed in monocytes/macrophages and fibrillar-type senile plaques in the cerebral cortex of AD patients (An et al., 2009). In addition, senile plaque formation promotes Lf deposition with age (Wang et al., 2010). Lipoprotein receptor-related protein (LRP) is a cell surface receptor involved in Aβ clearance via an endocytic process; Lf can bind to LRP and substantially enhance the clearance of soluble Aβ rather than the production of Aβ (Qiu et al., 1999). Recently, a liposomal system with surface Lf was developed in order to deliver neuron growth factor across the BBB. This method was shown to be beneficial in controlling the progression of AD (Kuo and Wang, 2014; Meng et al., 2015).

Another iron-binding protein, MTf, specifically localized in a subset of reactive microglia cells, and associated with the formation of senile plaques in the AD brain, was reported previously (Jefferies et al., 1996; Yamada et al., 1999). Although serum MTf levels are 3–4 fold higher in AD patients than in normal controls (Kim et al., 2001), other studies argued that serum MTf levels remained unchanged in AD patients and that MTf does not play a role in the transport of iron in the brain (Desrosiers et al., 2003; Suryo Rahmanto et al., 2012). Thus, further studies are now needed to investigate the role of MTf in the development of AD. In addition, an increase in heme oxygenase-1 level has been identified in astrocytes and neurons, resulting in co-localization with NFTs, senile plaques and corpora amylacea (Smith et al., 1994; Schipper, 2011; Gupta et al., 2014), suggesting that pathological iron deposition occurs due to the release of iron from heme proteins in AD-affected neural tissues.

### Copper

Copper is another essential transition metal that plays a critical role in several cellular functions; for example, as a structural component of enzymes required for energy metabolism and antioxidant defense. Furthermore, copper is involved in cellular respiration, free radical defense, and neurotransmitter synthesis (Zatta and Frank, 2007; Desai and Kaler, 2008; Scheiber and Dringen, 2013). Copper deficiency in the brain has an adverse effect on the development and maintenance of myelin and can induce degeneration of the nervous system. In contrast, excessive levels of copper also augment the Fenton reaction causing the generation of free radicals, as has been extensively reported for iron (Brewer, 2008; Gybina et al., 2009). Current evidence indicates that alteration in copper levels occurs in neurodegenerative diseases, such as AD.

The involvement of copper in the pathophysiology of AD is complex. High concentrations of copper have been detected in senile plaques (Lovell et al., 1998). In contrast, some studies have reported a deficiency of total copper brain levels in the AD brain (Deibel et al., 1996), and a recent meta-analysis showed that although the combined level of plasma and serum copper was higher in AD patients (Klevay, 2008), the total levels of copper in CSF were no different when compared between healthy subjects and AD patients (Ventriglia et al., 2012; Vaz et al., 2017). The rational explanation for this heterogeneity is that a significant quantity of copper precipitates with senile plaques in AD-affected regions, leading to copper deficiency in other regions. There is a common agreement that copper interacts with both Aβ and tau, and exacerbates their pathological consequences (Sparks and Schreurs, 2003; Kitazawa et al., 2009). Copper directly binds to Aβ with a high affinity and facilitates its oligomer formation (Tougu et al., 2008; Jin et al., 2011). The mechanism underlying copper-mediated Aβ oligomer cytotoxicity might involve oxidative stress, because copper and Aβ can catalytically generate hydrogen peroxide *in vitro*. The toxicity of the Cu-Aβ complex could be reversed by copper chelators, such as clioquinol (Matlack et al., 2014) and PBT2 (Adlard et al., 2008). In addition, APP and Aβ precursor-like protein 2 (APLP2) also have a copper binding site (Barnham et al., 2003). It has been reported that APP may function as a copper transporter since APP or APLP2 knockout mice showed elevated copper levels in the cerebral cortex (White et al., 1999b). However, the same author also published another study in which APP knockout in cortical neurons did not affect copper uptake (White et al., 1999a), indicating that APP may not be a copper-carrier, but rather shows inappropriate interactions with copper. Copper promotes the redistribution of APP at the cell membrane by enhancing exocytosis and reducing endocytosis (Acevedo et al., 2011). Moreover, copper increases the phosphorylation of endogenous APP via GSK3β and facilitates its proteolytic cleavage to generate Aβ (Acevedo et al., 2014). It was found that the microtubule-binding domain of tau can also bind with copper. Copper binds to tau and causes its aggregation *in vitro* (Su et al., 2007). In an *in vivo* mouse model of AD, copper exposure induced tau hyper-phosphorylation and generated hydrogen peroxide (Kitazawa et al., 2009). The mechanism of copper-mediated tau phosphorylation is thought to occur via the activation of CDK5 and GSK3β pathways (Crouch et al., 2009a).

**FIGURE 2:**
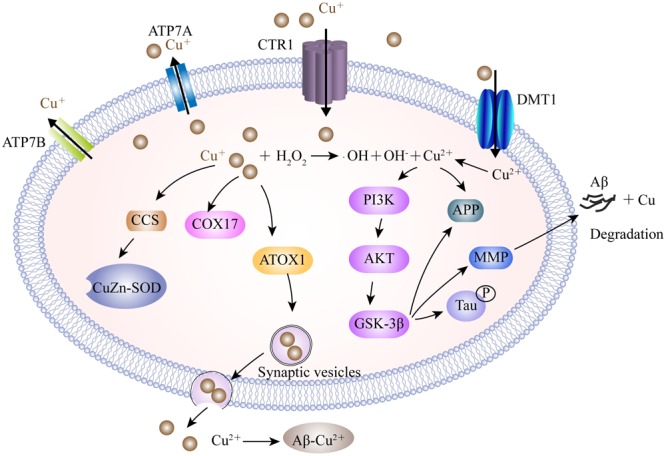
A model describing the copper transport system and its association with AD. Cu^+^ is taken up into brain cells by CTR1. DMT1 is involved in Cu^2+^ uptake. Accumulated Cu is sequestered into specific cellular locations by different Cu chaperones, such as CCS, COX17, and ATOX1. ATOX1 is suggested to transfer Cu^+^ to ATP7A and ATP7B, which help to import Cu^+^ into synaptic vesicles for release and/or directly mediate Cu export. Excessive intracellular Cu^+^ may activate the Fenton reaction to increase oxidative stress. Furthermore, Cu^2+^ is involved in the expression of the MMP responsible for the degradation of Aβ by activating the GSK3β pathway, which also contributes to tau hyper-phosphorylation. In the synaptic cleft, Cu binds to Aβ and facilitates the formation of senile plaques. CTR1, copper transporter 1; ATP7A, copper-transporting P-type ATPase; CCS, copper chaperone for superoxide dismutase; COX17, cytochrome oxidase enzyme complex; ATOX1, antioxidant protein-1; MMP, matrix metalloproteinases; GSK3β, glycogen synthase kinase 3β.

The mechanisms involved in copper dislocation in the AD brain remain clear. Copper transporter 1 (CTR1) and the copper transporting P-type ATPases, including ATP7A and ATP7B, are the major transporters involved in the cellular regulation of monovalent copper (Kuo et al., 2006; Yu et al., 2017) (**Figure 2**). DMT1 may participate in delivering divalent copper into cells for the synthesis of copper containing enzymes (Zheng and Monnot, 2012). Nevertheless, in the context of copper overload, ATP7A and ATP7B play dual-functions to export excess copper out of cells in a manner which is dependent on ATP hydrolysis. In addition to the transporters, a cluster of intracellular proteins, recognized as molecule chaperones such as antioxidant protein-1, cytochrome oxidase enzyme complex and copper chaperone for superoxide dismutase (SOD), also take part in delivering copper to specific targets (Harris, 2001; Zheng and Monnot, 2012). In a *Drosophila* model of AD, genetic knockdown of CTR1C, one of the CTR1 family members homologous to the human gene, significantly reduced copper accumulation in the brain (Lang et al., 2013). These consistent results were also observed in flies when CTR1B, another copper importer, was inhibited, or when DmATP7, a copper exporter, was enhanced in AD flies. These flies exhibited increased levels of Aβ production but a reduction in Cu-Aβ complex-induced oxidative stress, suggesting that Aβ oligomers, or the increased Aβ aggregates, were less toxic in a reduced copper influx mediated by CTR1 knockdown (Lang et al., 2013). In a mouse model of AD, ATP7A is upregulated in activated microglial cells where the amyloid plaques are clustered, resulting in a significant change of microglia copper trafficking. This depicts a neuromechanism in which inflammation-induced copper dyshomeostasis in microglia is associated with AD (Zheng et al., 2010). Genetic analysis of the genome of AD patients revealed that a cohort of single nucleotide polymorphisms in ATP7B account for imbalances in circulating nonceruloplasmin-bound copper which increase the risk of AD, supporting the notion that changes in copper homeostasis may accelerate the neurodegeneration that lead to AD (Bucossi et al., 2011; Bucossi et al., 2013; Squitti et al., 2013).

### Zinc

Zinc is the second most abundant metal in the body after iron and is a vital component of 100s of enzymes and proteins. Zinc is also involved in cell signaling in a much more extensive way than other metals, particularly because it can act as a neurotransmitter (Barr and Burdette, 2017). During neuronal activity, synaptic transmission releases zinc into the synaptic cleft, which features a major zinc-efflux mechanism in neurons. Zinc interacts with various post-synaptic receptors and transporters. For example, zinc inhibits *N*-methyl-D-aspartate receptors (NMDARs) and activates GPR39; collectively these mechanisms serve to modulate cell signaling and synaptic plasticity (Paoletti et al., 1997; Khan, 2016). Synaptic zinc deficiency not only affects the proper development of the brain, but also causes depression-like symptoms and dysfunctions in learning and memory (Swardfager et al., 2013). Furthermore, zinc deficiency has been reported to increase the overload of other metals, such as copper, nickel and possibly toxic metals, by upregulating zinc transporters (Antala and Dempski, 2012). However, excessive zinc can be sequestered by the mitochondria and triggers the generation of ROS by interfering with the activity of complex III of the electron transport chain, thereby promoting mitochondrial dysfunction and neuronal death in a manner which is relevant to a variety of disorders (Frazzini et al., 2006).

The role of zinc in the pathogenesis of AD has been intensively studied since a previous study described the redistribution of zinc into extracellular senile plaques (Bush et al., 1994). However, there is an inconsistent conclusion with regard to zinc levels in AD patients. Many studies have shown high levels of zinc in the AD brain and CSF (Religa et al., 2006; Hozumi et al., 2011). Some other reports reported no difference or even reduced zinc levels in the affected brain and serum of AD patients compared to controls (Panayi et al., 2002; Ventriglia et al., 2015; Wang et al., 2015). The reason for such heterogeneity in zinc levels remains unknown. Unfortunately, this is likely to hinder the development of ways to supplement or obliterate zinc as a form of AD treatment. Despite such conflict, the binding of zinc to histidine residues in the C-terminus of Aβ, and the promotion of aggregates, should be noted. Relative to iron and copper, zinc binds to Aβ with a greater affinity upon a wide range of pH (Yoshiike et al., 2001). On the one hand, this binding conceals the proteolytic cleavage site where Aβ is degraded by metalloproteases (Crouch et al., 2009b); on the other hand, binding induces the loss of zinc bioavailability in the synaptic cleft, thus contributing to changes in synaptic plasticity and a cognitive decline in AD (Deshpande et al., 2009). Furthermore, zinc deficiency may also have deleterious effects on the maturation of brain-derived neurotrophic factor (Khan, 2016). Although excessive zinc enhances Aβ toxicity, zinc also changes the conformation of Aβ and prevents copper from interacting with Aβ, which, in turn, ameliorates the oxidative stress burden (Cuajungco et al., 2000; Pedersen et al., 2012). In addition, zinc increases the expression of presenilin 1 in order to facilitate zinc uptake; at the same time, zinc also inhibits the activity of γ-secretase, which is involved in the generation of Aβ from APP (Hoke et al., 2005). Furthermore, zinc may also able to bind to tau proteins with moderate affinity and modulate phosphorylation by activation of GSK3β, extracellular regulated protein kinase 1/2 (ERK1/2), and c-Jun N-terminal kinase (JNK) (Pei et al., 2006; Lei et al., 2011).

**FIGURE 3:**
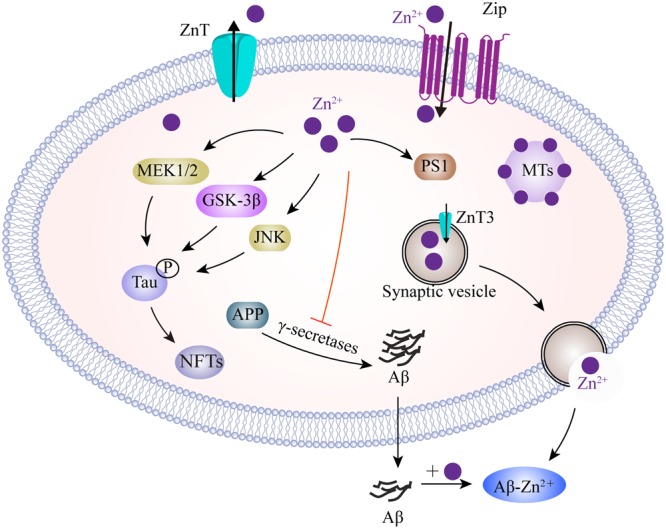
A model describing the zinc transport system and zinc imbalance in the AD brain. Zn^2+^ enters neurons, mainly depending on ZIPs, whereas Zn^2+^ efflux is controlled by ZnT in the plasma membrane. Intracellularly, MTs as the major Zn^2+^-buffering peptides, maintain Zn^2+^ at appropriate levels. In glutamatergic neurons, Zn^2+^ is transported into presynaptic vesicles by ZnT3. Thus, Zn^2+^ could be co-released with glutamate into the synaptic cleft during neuronal activity. Zn^2+^ binds to Aβ and promotes its aggregation. Increased Zn^2+^ enhances tau translation and phosphorylation by MEK1/2, GSK3β and JNK pathways. In addition, Zn^2+^ increases APP proteolysis, but inhibits γ-secretase activity. Zn^2+^ is also involved in the upregulation of PS1, which facilitates cellular Zn^2+^ uptake. ZIPs, zinc importing proteins; ZnT, zinc transporter; MTs, metallothioneins; MEK1/2, mitogen-activated protein kinase 1/2; GSK3β, glycogen synthase kinase 3β; JNK, c-Jun N-terminal kinase; PS1, presenilin 1.

In neurons, zinc homeostasis is principally controlled by three groups of transporters, comprising the zinc transporters (ZnTs), zinc-regulated transporter-like and iron-regulated transporter-like proteins (ZIPs), and metallothioneins (MTs) (**Figure 3**). ZnTs facilitate zinc efflux from cells or mediate excessive zinc from the cytoplasm into organelles and intracellular vesicles (Huang and Tepaamorndech, 2013). ZIPs play roles which are almost opposite to those of the ZnTs, and drive zinc import into cells or promote the movement of zinc from intracellular vesicles into the cytoplasm (Cousins et al., 2006; Huang and Tepaamorndech, 2013). MTs are zinc homeostasis-regulating proteins that control cellular zinc levels and related signaling pathways (Krezel et al., 2007). Immunofluorescent evidence demonstrated that ZnTs (ZnT1, 3, 4, 5, 6, and 7) are extensively present in Aβ plaques in the cortex of human AD brains (Zhang et al., 2008). Of these, ZnT3 is predominantly present on the synaptic vesicles of zinc-containing glutamatergic neurons (Palmiter et al., 1996). With aging, decreased levels of ZnT3 were found in elderly people, particularly in people with AD (Whitfield et al., 2014). ZnT3 knockout mice displayed an age-dependent deficit in cognition (Adlard et al., 2010), while mice overexpressing APP, but lacking ZnT3, appeared to have lower levels of synaptic zinc and a reduced plaque burden (Lee et al., 2002), implicating the contribution of synaptic zinc to the deposition of amyloid plaques in AD. In contrast, increased intra-neuronal zinc, caused by ZnT3 knockout, exacerbated neuronal damage in AD (Lovell et al., 1998). The reuptake of zinc into the presynapse still, however, requires characterization. The ZIPs have a fundamental role in mediating zinc influx, and other mechanisms are likely to indirectly participate, such as presenilins, mutations of which are known to cause familial AD (Greenough et al., 2011). In addition, MTs are major zinc-buffering peptides that maintain the cytosolic zinc balance. Four main MT isoforms (MT-1 to -4) are also expressed in brains. MT-1 and MT-2 are upregulated in AD patients, whereas MT-3 is downregulated (Hidalgo et al., 2006). In an animal model of AD, the lack of MT1/2 results in a reduction in the amyloid plaque burden, and therefore recovers the APP-induced changes in mortality (Manso et al., 2012a). MT3 is correlated to Aβ aggregation by cysteine oxidation. MT3 deficiency partially rescues the APP-induced mortality of females and leads to changes in APP-induced behavioral phenotypes of mice (Manso et al., 2012b). More recently, it was found that MT3 modulates Aβ uptake in astrocytes through its positive effect upon actin polymerization (Lee et al., 2015). Furthermore, a combination of microscopy and spectroscopy studies demonstrated that MT3 prevents Cu-Aβ-mediated neurotoxic effects probably via a metal exchange between Zn-MT3 and the aggregated Cu-Aβ (1-40), thus leading to the suppression of ROS production (Pedersen et al., 2012). In contrast, another study investigated the protective role of astrocyte-derived MTs on primary cortical neurons against Aβ toxicity by reducing ROS content, upregulating Bcl-2 and inhibiting proinflammatory cytokine production (Kim et al., 2012). To better understand AD-related zinc dyshomeostasis, it is necessary to investigate the role of zinc transporters in the etiology of this disease.

### Manganese

Manganese (Mn) is a critical trace element present in human tissues and exerts important physiological roles for growth and intracellular homeostasis (Prakash et al., 2016). Manganese is also used as a cofactor for key enzymes involved in normal cell function, such as SOD and glutamine synthetase. An increasing number of reports have provided evidence that Mn overload is associated with neurodegenerative diseases, and that even a small excess of Mn can induce symptoms that are consistent with manganism (Park, 2013). The mechanisms of Mn-mediated cytotoxicity involve the over-production of ROS, mitochondrial dysfunction, abnormal energy metabolism, accumulation of intracellular toxic metabolites, the depletion of cellular antioxidant defense and autophagy (Guilarte, 2013; Martinez-Finley et al., 2013).

Manganese was detected in significantly higher levels in the brain of AD patients with dementia compared to healthy subjects, while the highest level of Mn was detected in the parietal cortex (Srivastava and Jain, 2002; Tong et al., 2014). This suggests that Mn overload may be involved in the pathology of AD and cognitive dysfunction. Chronic Mn exposure to non-human primates was shown to alter gene expression which dispersed Aβ plaques. Most of the altered genes were targeted by p53; one such gene was amyloid-beta precursor-like protein 1 (APLP1), the most highly upregulated gene in the frontal cortex (Guilarte, 2010). Mn exposure appears to particularly target the frontal cortex leading to incipient dementia (Schneider et al., 2013). In addition, Mn treatment increases levels of Aβ peptides both *in vitro* and *in vivo*; the mechanism involved is probably related to the disruption of Aβ degradation (Tong et al., 2014). More recently, a study showed that Mn could weakly bind to the specific site of Aβ, as described for other biometals (Wallin et al., 2016). However, the effects of such binding by Mn to Aβ in the promotion of Aβ aggregation need to be further demonstrated. Mn is a component of Mn superoxide dismutase (Mn-SOD), which is an antioxidant enzyme that plays an important role in maintaining mitochondria vitality. Increased Mn obstructs oxidative respiration, thus increasing ROS production and eventually leading to mitochondrial dysfunction (Gunter et al., 2006). Partial deficiency of Mn-SOD increased Aβ plaque deposition and tau phosphorylation in a transgenic mouse model of AD (Li et al., 2004; Melov et al., 2007). In contrast, the overexpression of Mn-SOD showed some benefit against AD pathology by reducing the burden associated with cortical plaques (Dumont et al., 2009), which further demonstrated the links between mitochondrial oxidative stress and the pathophysiology of AD. In addition, Mn toxicity results in a cognitive decline in humans, and it has been postulated that excessive Mn uptake causes iron deficiency in the Golgi apparatus, consistent with fact that Mn and iron compete with the same binding sites and transport mechanisms, at least to some extent (Carmona et al., 2010).

**FIGURE 4:**
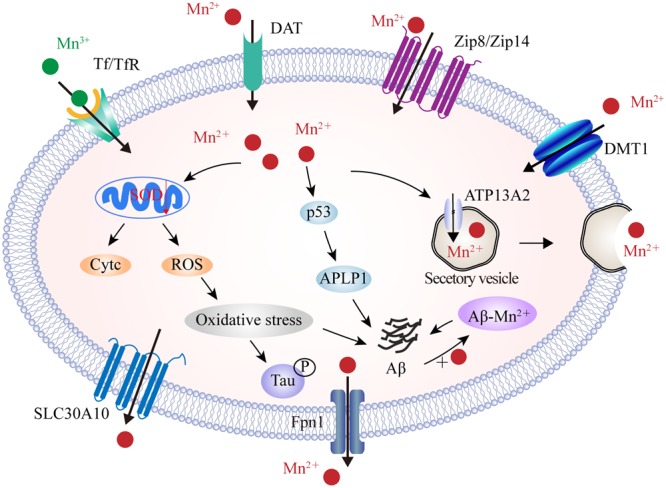
The identified manganese transport system and its association with AD. On the cell membrane, DMT1, ZIP8/ZIP14, and DAT are responsible for Mn^2+^ influx, while Tf/TfR mediates Mn^3+^ entry into the endosome through endocytosis and is eventually released into the cytoplasm by DMT1. In contrast, SLC30A10 and Fpn transport Mn^2+^ out of cells. Furthermore, ATP13A2 and SPCA1 also transport Mn^2+^ into the lysosomes and Golgi for bioavailability or by forming secretary vesicles that facilitate Mn^2+^ efflux. In the AD brain, Mn^2+^ exposure induces mitochondrial oxidative stress that further advances tau phosphorylation. Moreover, high Mn^2+^ levels increase the expression of p53 and its transcriptional target gene, APLP1, which consists of APP. Increased APLP1 expression promotes the generation of Aβ peptides. Mn^2+^ could also bind to Aβ and enhance its aggregation. DAT, dopamine transporter; SLC30A10, solute carrier family 30 member 10; ATP13A2, ATPase 13A2; SPCA1, secretory pathway Ca^2+^-ATPase 1; APLP1, amyloid-b precursor-like protein 1.

Manganese transport is mediated by multiple importers, including DMT1, Tf/TfR, ZIP8 and ZIP14, and dopamine transporter (DAT), as well as by various exporters including park9/ATP13A2, SLC30A10, Fpn, and the secretory pathway Ca^2+^-ATPase 1 (SPCA1) (**Figure 4**). Of these, DMT1 is not only a transporter for iron influx, but also the first mammalian transporter for cellular Mn uptake. DMT1 facilitates the movement of Mn across the BBB, particularly under in conditions of iron scarcity (Garrick et al., 2006). DMT1 transports divalent Mn, while Tf transports trivalent Mn into cells through a ligand-receptor endocytosis mechanism (Subramaniam et al., 2002). ZIP8 and ZIP14 have high binding ability for zinc, but several studies have found that they are also involved in the absorption of body Mn from the liver and lungs (Fujishiro et al., 2012; Bekerydemir et al., 2017; Lin et al., 2017). In recent years, efforts have focused upon the role of exporter proteins in maintaining Mn levels. Genome analysis identified SLC30A10, a cell surface-localized efflux exporter, which may transport both zinc and Mn. Patients who suffer from Parkinson’s disease, and carry mutations in SLC30A10, show accumulation of Mn in the brain (Tuschl et al., 2016). SLC30A10 has consistently shown to be significantly reduced in the frontal cortex of AD patients, and in APP/PS1 transgenic mice, suggesting that its dysregulation contributes to the pathology of AD (Bosomworth et al., 2013). A number of studies have also shown that the iron exporter Fpn could also function as a cellular exporter of Mn in a pH-dependent manner in order to attenuate Mn accumulation and cytotoxicity (Madejczyk and Ballatori, 2012; Seo and Wessling-Resnick, 2015). Furthermore, ATP13A2 acts as a cation transporter in Mn and zinc transportation. Studies have shown that the over-expression of ATP13A2 reduces intracellular Mn concentration, which in turn, alleviates Mn-provoked lethality; loss-of-function mutations in ATP13A2 are correlated with increases in both α-synuclein and Aβ plaques in Lewy body disease (Murphy et al., 2013). Moreover, plasma membrane ATPase-related 1 (PMR1), a SPCA1 homolog in yeast, has been shown to mediate Ca and Mn transport, and the ectopic expression of SPCA1 in yeast increases sensitivity to Mn toxicity (Ton et al., 2002). Thus, it has been suggested that SPCA1 represents another regulator for cellular Mn homeostasis, although the affinity between SPCA1 and Mn, as well as the roles of SPCA1 in AD pathogenesis, require further investigation.

### Magnesium and Calcium

Magnesium (Mg) is one of the most abundant divalent cations in cells and has been known to be required for multiple enzymatic synthesis and cellular processes including energy metabolism, ion channels and synaptic plasticity (Misra and Draper, 1998; Bairoch, 2000). Calcium (Ca) acts as a ubiquitous second messenger that has been studied extensively with regard to its role in controlling cellular function (Komuro and Kumada, 2005). Intracellular Ca concentrations are tightly regulated by various Ca channels, pumps and Ca binding proteins, and are also modulated by other metal ions, such as Mg. In neurons, Mg and Ca collectively play a vital role in neuronal growth and signal transmission. It is noteworthy that Mg has been identified as an antagonist to Ca. Under physiological conditions, Mg maintains intracellular Ca levels and protects neurons from excitatory responses induced by Ca overload (Levitsky and Takahashi, 2013). However, disturbances in Mg and Ca homeostasis alter a cascade of events which lead to a variety of diseases including diabetes, cancer, and neurodegeneration (Volpe, 2013).

There is compelling evidence that brain and serum Mg levels are significantly lower, and Ca levels significantly higher, in AD patients than those in age-matched healthy subjects (Andrasi et al., 2005; Cilliler et al., 2007; Stutzmann, 2007). Chronically increased Ca levels increase the expression of APP and ApoE and facilitate the formation of Aβ aggregation through a mechanism involving the stabilization of γ-secretase (Brzyska and Elbaum, 2003; Ho et al., 2010), and reciprocally, Aβ aggregation alters membrane Ca permeability that further worsens AD (Kelly and Ferreira, 2006). Few studies have investigated the roles of Mg in the pathogenesis of AD. An *in vitro* study showed that both Ca and Mg can trigger the process of hyper-phosphorylated tau aggregation (Yang and Ksiezak-Reding, 1999). The administration of magnesium-l-threonate increases Mg levels in the brain, which decreases levels of β-secretase (BACE1) thus reducing the levels of soluble APP and β c-terminal fragments, thus alleviating synaptic loss and the cognitive deficits associated with Alzheimer’s symptoms (Li et al., 2014). Furthermore, treatment with Mg sulfate reduces levels of hyper-phosphorylated tau by inhibiting GSK-3β phosphorylation and increasing the activity of protein kinase B (Akt) and phosphatidylinositol 3 kinase (PI3K) (Gomez-Ramos et al., 2006; Xu et al., 2014), thus suggesting that Mg acts as a neuroprotective factor in the development of AD. It is though that the underlying mechanism involves Mg blocking the long term activation of NMDAR-mediated Ca influx and thus reducing Ca-induced neuroinflammation. NMDARs are cationic channels activated by glutamate, possessing a high permeability to Ca ions upon synaptic activity, such as learning and memory. It has been shown that the over-activation of NMDAR by Aβ aggregation may occur during the early stages of AD (Parameshwaran et al., 2008). Continuous Ca influx increases intracellular Ca levels, which initiates a number of enzymatic processes that result in protein destruction, peroxidation, and neuronal death (Mota et al., 2014). Under normal conditions, Mg, as an endogenous blocker, binds to NMDAR subtypes, NR1/2A and NR1/2B, which are constituent parts of NMDARs present in AD-affected brain regions (Kotermanski and Johnson, 2009). Blocking channels by adding Mg reduces Ca influx into post-synaptic neurons, so as to alleviate excitotoxic cell death during dementia. In addition, the activation of ATP-gated P2X purinergic receptors (P2XRs) associated with neuroinflammation has been identified in neurodegenerative disease (Witting et al., 2004). One subtype, P2X7R, can form an oligomer to create membrane pores in microglia, thus facilitating Ca influx (North, 2002). Using tissue culture, it has been shown that Mg can reduce levels of intracellular Ca through P2X7R, and that ameliorated purinergic receptor stimulation activated neuroinflammation, suggesting that elevated Mg is a broad spectrum inhibitor of Ca entry through cell surface channels (Lee et al., 2011).

**FIGURE 5:**
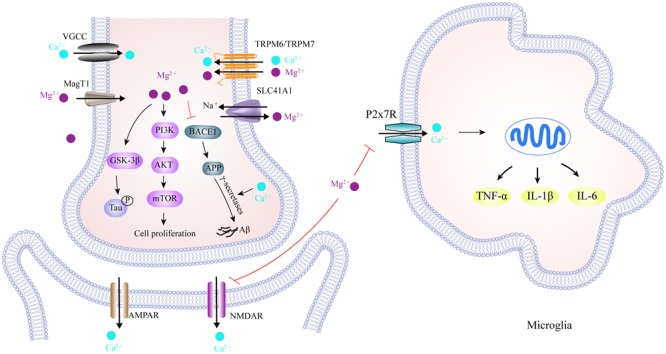
Scheme of magnesium and calcium transporters and their interaction in the AD brain. TRPM6/TRPM7, and MagT1, located in the cell membrane, facilitate Mg^2+^ influx, while SLC41A1 functions as a Na^+^/Mg^2+^ exchanger and mediates Mg^2+^ efflux. Meanwhile, cellular Ca^2+^ transport is mediated by VGCC, NMDAR and AMPAR, as well as P2X7R in the microglia. In the cytoplasm, activation of the PI3K/Akt/mTOR signaling pathway by Mg^2+^ is required for cell growth, proliferation and the inhibition of autophagy. An increase in Mg^2+^ levels down-regulates BACE1 expression, which decreases Aβ production from APP, whereas Ca^2+^ stabilizes γ-secretase to promote Aβ formation. It has also been reported that Mg^2+^ reduces tau hyper-phosphorylation by inhibiting GSK3β. More importantly, increased Mg^2+^ can block NMDAR-mediated Ca^2+^ influx in the post-synapse and P2X7R-mediated Ca^2+^ influx in microglia, thus alleviating the excitotoxicity and neuroinflammation caused by excessive Ca^2+^ influx in AD brain. TRPM6/TRPM7, transient receptor potential melastatin 6 and 7; MagT1, magnesium transporter 1; SLC41A1, solute carrier family 41 member 1; VGCC, voltage-gated Ca^2+^ channels; NMDAR, *N*-Methyl-D-Aspartic acid receptor; GSK3β, glycogen synthase kinase 3β; AMPAR, α-amino-3-hydroxy-5-methyl-4-isoxazolepropionic acid receptor; BACE1, beta-secretase 1; P2X7R, purinergic P2X7 receptor.

Over recent decades, a large body of transporters, channels, exchangers, and buffering proteins have been found to be involved in the maintenance of cellular Mg and Ca homeostasis. As illustrated in **Figure 5**, a range of channels are known to mediate Mg influx into cells, including magnesium transporter 1 (MagT1) and transient receptor potential melastatins 6 and 7 (TRPM6/TRPM7), as well as cyclin M (CNNM) transporter. Correspondingly, sodium-independent Mg exchanger and solute carrier family 41 member 1 (SLC41A1) are required to favor Mg extrusion (Romani, 2011; de Baaij et al., 2015). Furthermore, intracellular Ca balance can be achieved through a large number of Ca transporters. Voltage-gated Ca channels, store-operated Ca channels, NMDAR, and α-amino-3-hydroxy-5-methyl-4-isoxazolepropionic acid receptors (AMPARs) are responsible for increases in Ca. Ca-binding buffering proteins, such as calbindin, facilitate the storage of Ca in the endoplasmic reticulum (ER), whereas the action of the Na/Ca exchanger, as well as the Ca-ATPase pump, mediates the export of Ca out of cells. In the AD brain, mutant presenilins are known to activate two types of Ca receptors and that plasma membrane Ca-permeable channels allow the leakage of Ca ions from the ER into the cytoplasm, thus causing significant effect upon ER-Ca dynamics (Tu et al., 2006; Cheung et al., 2008). Moreover, studies have indicated that Aβ oligomers can either stimulate the formation of Ca permeable channels, or bind to NMDARs, thus promoting Ca entry through the plasma membrane (Diaz et al., 2009; Arbel-Ornath et al., 2017). However, our knowledge about the roles of Mg transporters in AD pathology is very limited. Recently, the physiological role of TRPM7 was found to be coordinated by presenilins, the mutation of which can lead to Familial Alzheimer’s disease (Oh et al., 2012). Elimination of TRPM2 in APP/PS1 mice reduced ER stress and improved age-dependent memory deficits, while *in vitro* studies showed that the knockdown of TRPM2 blocked the Aβ-mediated increase in whole cell current magnitude, thus indicating the importance of TRPM2 activity in Aβ neuronal toxicity (Ostapchenko et al., 2015). In this study, the authors reported that TRPM2 alteration leads to Ca imbalance, although its role in the regulation of Mg associated with AD was ignored (Ostapchenko et al., 2015). In addition, Amyotrophic Lateral Sclerosis and Parkinsonism Dementia has also been shown to be associated with lower Ca and Mg levels than healthy subjects, thus implicating the dysfunction of TRPM2 and TRPM7 channels (Hermosura and Garruto, 2007).

### Non-biologically Relevant Metals and its Toxicity

#### Aluminum

Aluminum (Al) is a toxic metal to organisms with unknown physiological roles. We inevitably contact Al in daily life due to its ubiquitous presence in the environment. Fortunately, much of our dietary intake of Al compounds is not dissolved at physiological pH but rather, is subsequently excreted out of the body. Al toxicity only occurs when high levels of Al are ingested or inhaled. In the brain, Al accumulates mainly in the hippocampus and frontal cortex, thus correlating to the dysregulation of other essential biometals. This leads to oxidative damage and affects a large number of signaling cascades, features which can lead to neuronal death and the induction of neurodegenerative diseases (Kawahara and Kato-Negishi, 2011; Wu et al., 2012; Nampoothiri et al., 2015).

Previous literature reported the aluminum hypothesis, which hinted that Al exposure is involved in the etiology of AD (Klatzo et al., 1965; Crapper et al., 1973). Although the authenticity of the aluminum hypothesis in AD has been debated for decades, and is gradually being considered as only a fringe theory in comparison to a number of other theories in AD research, Al exposure still remains attractive and continues to be the focus of interest (Lidsky, 2014). In an *in vivo* study, chronic Al administration to rats resulted in an increase in Aβ production in the hippocampus and cortical regions (Fattoretti et al., 2004). Al administration to a transgenic mice model of AD also accelerated the accumulation of Aβ plaques and enhanced amyloidogenesis, although this effect could be eliminated by antioxidant treatment (Pratico et al., 2002). These data indicated that the neurotoxic effect of Al results in increased oxidative stress. These results were also seen in cultured neurons, in which long-term exposure to Al showed Aβ aggregation and fibrillar deposits on the surface of cells (Exley et al., 1993; Kawahara et al., 2001). Furthermore, high levels of Al in the body affected the roles of three important genes, APP, presenilin-1, and presenilin-2 (Sato et al., 1999). Other studies have reported that Al reduced the activity of some key enzymes related to Aβ catabolism by activating the amyloidogenic pathway (Sakamoto et al., 2006; Liang et al., 2013), implicating a possible reduction in Aβ degradation. Furthermore, Al accelerates the aggregation of hyper-phosphorylated Tau protein by inhibiting protein phosphatase 2A activity (Yamamoto et al., 1990). Although Al-loading induces neurotoxic effects, and produces behavioral changes that partially model AD, the indication of whether toxic Al exposure acts as a causative factor for AD still remains to be verified.

The molecular mechanisms associated with Al transport in neurons remain unclear. There is now considerable evidence to show that Al transport and uptake into cells is somewhat complicated by other metal ions, such as iron, illustrating that Al competes with iron in terms of binding to iron transporters, Lf/LfR or Tf/TfR, which also transport Al across the BBB (Yokel, 2006). In *Caenorhabditis elegans*, SMF-3, a homolog of human DMT1, was found to transport Al into neurons, leading to excessive levels of Al which reduced the mitochondrial membrane potential and cellular ATP levels (VanDuyn et al., 2013). Al also inhibits iron-induced oxidation and the degradation of iron regulatory protein 2 (IRP2) through a ubiquitin-proteosomal pathway, suggesting that Al stabilizes IRP2 to interfere with intracellular iron balance (Yamanaka et al., 1999). Noticeably, Al-induced neurodegeneration appears to be associated with a different molecular pathway that is independent of Aβ- or tau-associated toxicity, and is mostly arbitrated by iron accumulation and ROS production in the brain (Wu et al., 2012).

#### Other Toxic Metals

Lead (Pb) is a household neurotoxin which causes mental retardation, neuronal loss, and neurotransmission failure. However, the primary risk of Pb toxicity is its ability to bind to sulfhydryl groups, in which it can influence a variety of enzymic activities. Furthermore, Pb may mimic physiological metals and compete for their binding sites (such as Ca and zinc) to disrupt corresponding biometal-dependent mechanisms (Kern et al., 2000; Crumpton et al., 2001). A range of studies have reported that Pb exposure in infants is associated with cognitive deficits and behavioral disturbance, and that Pb exposure in pregnant women increases the possibility of low birth rate due to spontaneous abortions (Borja-Aburto et al., 1999; Dorsey et al., 2006).

Although epidemiological studies have reported that serum Pb has no causal correlation with AD (Park et al., 2014), a number of experimental research studies have provided evidence to suggest that Pb exposure could advance the formation of biomarkers in AD. In an *in vitro* study, SH-SY5Y cells treated with Pb displayed a significant reduction in the mRNA and protein levels of DNA methylating enzymes, which resulted in increased APP expression and reduced Aβ degradation (Huang et al., 2011; Bihaqi and Zawia, 2012). Another study showed that neonatal rats exposed to Pb showed an elevation in APP expression and its amyloidogenic Aβ product. In contrast, the relevant pathology exhibited a delayed response to Pb exposure during old age (Basha et al., 2005). Moreover, Pb exposure in mice reduced the expression of lipoprotein receptor-related protein 1 (LRP1), as a result of increased Aβ accumulation in the CSF, hippocampus, and frontal cortex (Gu et al., 2011). The long-term exposure of Pb in juvenile primates also resulted in high Aβ plaque levels in the brain (Wu J. et al., 2008). Collectively, these data provide evidence that Pb poisoning during brain development can increases the risk of AD.

Cadmium (Cd) is a widespread heavy metal that is released from industrial wastes. Cd toxicity is associated with kidney dysfunction, inactivation of the enzymatic system, the disruption of intracellular Ca homeostasis, and apoptosis (Yuan et al., 2013). In addition, Cd can cross over the BBB and long-term exposure to Cd causes its accumulation in the brain which can activate various signaling cascades to stimulate inflammation, oxidative stress, and lead to neuronal death, which eventually influences olfaction, attention, and cognitive function (Figueiredo-Pereira et al., 1998; Wang and Du, 2013).

A large number of clinical and experimental investigations have also suggested that Cd neurotoxicity is linked to the pathogenesis of AD (Del Pino et al., 2014). A previous study of AD patients with dementia showed that an increased Cd level was detected in plasma, while a reduced level was detected in the CSF (Basun et al., 1991). However, subsequent studies found that there was no correlation between blood Cd and dementia in AD (Basun et al., 1994). Other studies have reported no obvious differences in the plasma, CSF and some important brain regions for cognition, such as the hippocampus and amygdala, in subjects with AD (Tandon et al., 1994; Gerhardsson et al., 2008; Akatsu et al., 2012). In fact, the levels of Cd in serum are age-dependent, both in AD patients and normal individuals (Park et al., 2014). Although the variable detection of Cd levels, Cd exposure and its association with Aβ production have been well-described. A pulse-chase study showed that cadmium chloride increased APP levels but dramatically reduced APP proteolysis, while APP mRNA levels remained unchanged in cultured cells following cadmium chloride treatment (Smedman et al., 1997). In APP/PS1 transgenic mice, Cd treatment aggravated memory loss, which was accompanied by increases in the number and size of senile plaques, as well as in the overproduction of Aβ (1-42) in the cortex and hippocampus (Li et al., 2012). A subsequent study further demonstrated that Cd could interact with Aβ (1-42) by acting on a membrane-incorporated ion channel to facilitate Aβ aggregation (Notarachille et al., 2014). Additionally, Cd could promote the conformational change and self-aggregation of tau protein by binding to the third repeat of its microtubule-binding domain (Jiang et al., 2007). Collectively, these data reinforce the hypothesis that Cd is an etiological factor responsible for the development of AD.

Mercury (Hg) is another heavy metal that is of great public concern due to its extreme toxicity. Methylmercury (MeHg), the major environmental source of organic Hg, is directly produced by industrial processes and indirectly formed by the action of microbes that live in the soil and rivers. Exposure to MeHg represents a hazard for brain development, and can induce several symptoms, including sensory impairment, cognitive dysfunction, and motor disabilities (do Nascimento et al., 2008). In addition, the long-term or acute exposure of mercury vapor can result in the impairment of pulmonary function and psychotic reactions, as well as neurodegeneration (Liu et al., 2003; Cercy and Wankmuller, 2008).

The correlation of Hg and AD has been examined in a variety of animal models and *in vitro* studies. A number of studies have shown that Hg levels are increased in the blood and various brain regions in AD patients with major depression as compared to control subjects (Cornett et al., 1998; Hock et al., 1998). In cultured SH-SY5Y cells, both Aβ (1-40) and Aβ (1-42) secretion and phosphorylated Tau levels were significantly increased by HgCl_2_ treatment. The cytotoxicity of Hg is concomitant with increased ROS production, implicating the effects of Hg upon oxidative stress (Olivieri et al., 2000). Similar results were obtained in PC12 cells showing that MeHg treatment increased Aβ (1-40) accumulation through the over-production of APP as well as the reduction of neprilysin-mediated Aβ degradation (Song and Choi, 2013). In comparison with the effects of Pb, the accumulation of Hg increased Aβ and APP levels but did not result in changes in neprilysin activity, indicating that Hg and Pb might increase Aβ levels by different mechanisms (Chin-Chan et al., 2015b). Furthermore, MeHg exposure in rats resulted in the reduced expression of low-density LRP1 and an increased expression of the receptor for advanced glycation end products (RAGEs) in the brain capillary endothelium. These results revealed another mechanism in that MeHg-induced Aβ accumulation in the brain appeared to be mediated by disruption of its own transport (Kim et al., 2014). The administration of MeHg also induced Tau hyper-phosphorylation in the cerebral cortex by a selective manner through the activation of JNK pathways (Fujimura et al., 2009). Therefore, these studies provide evidence to indicate that Hg, in the same manner as Pb and Cd, contributes to the pathogenesis of AD. Further research is now warranted to elucidate the mechanisms underlying the neurotoxicity of toxic metals in AD.

## Conclusion

Based on current studies, it is likely that imbalance in intracellular biometal homeostasis and toxic metal exposure plays a contributory role in the pathology, and possibly the etiology, of AD. This article reviews the influence of various biometals in the main pathological hallmarks of AD. Five biometals (iron, zinc, copper, manganese, and calcium) are known to be deposited in the brains of AD subjects, as a result of the increased expression of APP, Aβ plaques aggregation, and tau hyper-phosphorylation. Exposure to toxic metals (aluminum, lead, cadmium, and mercury) may also initiate a characteristic pathology of AD via mechanisms including oxidative stress, neuroinflammation, and protein modification. In addition, the administration of magnesium has implied a neuroprotective role in animal models of AD. A complex network of transporters that mediate metal import or export appears to be involved in the development of AD (**Table 1**). However, some transporters are not specific for one metal as they can also transport other metals/substrates. For example, DMT1 plays a role in the uptake of both iron and manganese, indicating a synergistic effect in altered multiple metal homeostasis mediated by the dysregulation of transporters or metal-related proteins. Thus, further studies are now needed to explore broader changes of combined metal ion homeostasis in AD. Finally, this review raises awareness that intracellular metal dyshomeostasis can induce AD pathology and that metal metabolism-related proteins are appealing targets for therapeutic interventions.

**Table 1:** Dysregulation of different types of metal ion transporters in the AD brain.

Biometal	Transporters	Expression	Biological functions	Reference
Iron	DMT1	↑	Transport Fe^2+^, Cu^2+^, Mn^2+^ into cytosol	Zheng et al., 2009
	Tf/TfR	↑	Tf/TfR is endocytosed, by which Fe^3+^ is reduced to Fe^2+^	Moos and Morgan, 2000
	Lf/LfR	↑	Lf/LfR complex facilitate Fe^3+^ uptake	An et al., 2009
	Fpn	↓	Mediate Fe^2+^, Mn^2+^ export out of cells	Xian-hui et al., 2015
	MTf		Transport Fe^3+^ across the BBB	Kim et al., 2001
Copper	CTR1		Cell surface Cu^+^ uptake	Lang et al., 2013
	ATP7A	↑	Cellular Cu^+^ exporter	Zheng et al., 2010
	ATP7B	↑	Cellular Cu^+^ exporter	
Zinc	ZnT3	↓	Export Zn^2+^ from neurons	Adlard et al., 2010
	ZIPs		Zn^2+^ uptake	Greenough et al., 2011
	MT1, MT2	↑	Expressed in astrocytes for Zn^2+^ uptake	Hidalgo et al., 2006
	MT3	↓	Expressed in neurons for Zn^2+^ uptake	
Manganese	ZIP8,ZIP14		Mn^2+^ influx into the cytosol	Bekerydemir et al., 2017; Lin et al., 2017
	DAT		Mn^2+^ influx into the cytosol	Chen et al., 2006
	ATP13A2	↓	Transport Mn^2+^ into the lysosomes	Murphy et al., 2013
	SLC30A10	↓	Mediate efflux of Mn^2+^	Tuschl et al., 2016
	SPCA1		Transport Mn^2+^ into the Golgi	Ton et al., 2002
Magnesium	MagT1		Mediate Mg^2+^ influx	Goytain and Quamme, 2005
	TRPM6/TRPM7	↑	Mg^2+^ entry channel	Sun et al., 2015
	SLC41A1		Mediate Na^+^-dependent Mg^2+^ extrusion	Kolisek et al., 2012
Calcium	VGCC		Control Ca^2+^ influx under electrical activity	Thibault et al., 2007
	Na^+^/Ca^2+^ exchanger		Extrude Ca^2+^ from the cytoplasm in exchange for Na^+^ entry	Wu M.P. et al., 2008
	NMDAR	↑	Mediate Ca^2+^ influx	Birnbaum et al., 2015
	AMPAR	↑	Mediate Ca^2+^ influx	Whitehead et al., 2017
	P2X7R	↑	Mediate Ca^2+^ influx	Lee et al., 2011

## Author Contributions

YL wrote the manuscript; QJ, HX, XD, LS, FJ, and HJ approved and revised the final manuscript.

## Conflict of Interest Statement

The authors declare that the research was conducted in the absence of any commercial or financial relationships that could be construed as a potential conflict of interest.
